# Barriers and facilitators to the implementation of a new European eHealth solution (SurPass v2.0): the PanCareSurPass Open Space study

**DOI:** 10.1007/s11764-023-01498-8

**Published:** 2023-11-28

**Authors:** Ismay A. E. de Beijer, Emma C. Hardijzer, Riccardo Haupt, Desiree Grabow, Julia Balaguer, Edit Bardi, Adela Cañete Nieto, Audronė Ciesiūniene, Vanessa Düster, Anna-Liesa Filbert, Hannah Gsell, Monika Kapitančukė, Ruth Ladenstein, Thorsten Langer, Monica Muraca, Selina R. van den Oever, Sofie Prikken, Jelena Rascon, Maria Teresa Tormo, Anne Uyttebroeck, Gertrui Vercruysse, Helena J. H. van der Pal, Leontien C. M. Kremer, Saskia M. F. Pluijm, the PanCareSurPass consortium

**Affiliations:** 1 Princess Máxima Center for Pediatric Oncology, Heidelberglaan 25, 3584 CS Utrecht, The Netherlands; 2 IRCCS Istituto Giannina Gaslini, Genoa, Italy; 3 Division of Childhood Cancer Epidemiology, German Childhood Cancer Registry, Institute of Medical Biostatistics, Epidemiology and Informatics (IMBEI), University Medical Center, of the Johannes Gutenberg University Mainz, Mainz, Germany; 4 Hospital Universitario y Politécnico La Fe, Valencia, Spain; 5 St. Anna Children’s Hospital, Vienna, Austria; 6 Department of Paediatrics and Adolescent Medicine, Johannes Kepler University Linz, Kepler University Hospital, Linz, Austria; 7 Vilnius University Hospital Santaros Klinikos, Vilnius, Lithuania; 8 St. Anna Children’s Hospital and Children’s Cancer Research Institute, Department of Studies and Statistics for Integrated Research and Projects, Department of Paediatrics, Medical University of Vienna, Vienna, Austria; 9 CCI Europe, Vienna, Austria; 10 Universitatsklinikum Schleswig-Holstein, Campus Lubeck, Lübeck, Germany; 11 University Hospitals Leuven, KU Leuven, Louvain, Belgium; 12 Instituto de Investigación Sanitaria La Fe, Valencia, Spain; 13 University Medical Center Utrecht, Wilhelmina Children’s Hospital, Utrecht, the Netherlands; 14 Department of Pediatrics, Emma Children’s Hospital, Amsterdam UMC, University of Amsterdam, Amsterdam, The Netherlands

**Keywords:** Paediatric oncology, Long-term follow-up care, Survivorship care, Survivorship Passport, SurPass, eHealth, Open Space Technology

## Abstract

**Purpose:**

To identify barriers and facilitators for implementing the Survivorship Passport (SurPass) v2.0 in six long-term follow-up (LTFU) care centres in Europe.

**Methods:**

Stakeholders including childhood cancer survivors (CCSs), healthcare providers (HCPs), managers, information and technology (IT) specialists, and others, participated in six online Open Space meetings. Topics related to Care, Ethical, Legal, Social, Economic, and Information & IT-related aspects of implementing SurPass were evaluated.

**Results:**

The study identified 115 barriers and 159 facilitators. The main barriers included the lack of standardised LTFU care in centres and network cooperation, uncertainty about SurPass accessibility, and uncertainty about how to integrate SurPass into electronic health information systems. The main facilitators included standardised and coordinated LTFU care in centres, allowing CCSs to conceal sensitive information in SurPass and (semi)automatic data transfer and filing.

**Conclusions:**

Key barriers to SurPass implementation were identified in the areas of care, ethical considerations, and information & IT. To address these barriers and facilitate the implementation on SurPass, we have formulated 27 recommendations. Key recommendations include using the internationally developed protocols and guidelines to implement LTFU care, making clear decisions about which parties have access to SurPass data in accordance with CCSs, and facilitating (semi)automated data transfer and filing using Health Level 7 (HL7) Fast Healthcare Interoperability Resources (FHIR).

**Implications for Cancer Survivors:**

The findings of this study can help to implement SurPass and to ensure that cancer survivors receive high-quality LTFU care with access to the necessary information to manage their health effectively.

**Supplementary Information:**

The online version contains supplementary material available at 10.1007/s11764-023-01498-8.

## Introduction

Approximately 400,000 children and adolescents across the globe develop cancer each year [1]. Fortunately, due to the constant improvement of cancer treatments, the 5-year survival rate for childhood cancer in high-income countries is now above 80% [1, 2]. In Europe, there are around 500,000 childhood and adolescent cancer survivors (CCSs), with circa 8000–10,000 new CCSs being added each year [3, 4]. Unfortunately, at least 75% of CCSs develop adverse health effects after treatment [5, 6]. These late health effects may include, for example, organ dysfunction, subsequent (malignant) neoplasms and cognitive problems. Because CCSs often receive long and intensive treatment with chemotherapy and radiotherapy during a crucial period of physical and mental development and because the number of life years within which subsequent complications can develop is longer, CCSs are more likely to develop adverse health effects than adult survivors [7, 8]. Late health effects are particularly troublesome because they may lower the quality of life and even increase the risk of early mortality [5–10].

Long-term follow-up (LTFU) is essential for the early detection, prevention, and treatment of late health effects, thereby reducing their burden. Optimal LTFU care includes regular surveillance and treatment of late health effects, education about prevention and self-care of late effects, and encouragement of a healthy lifestyle for CCSs well beyond their paediatric age [11–13]. LTFU care programmes can vary between countries and even between centres and are usually tailored to the primary diagnosis and treatment of CCSs during their cancer period [14, 15]. During the last decade, person-centred care has come to play an important role in high-quality LTFU care standards [16].

Person-centred care considers all physical, mental, and social health needs of CCSs and encourages shared decision-making between HCPs and patients [11, 12, 16–18]. However, this approach faces several challenges, including a lack of awareness among CCSs of their need for LTFU care [19, 20], a lack of information about late health effects among HCPs [14, 21], and an unmet need for personalised treatment summaries and survivorship care plans [10, 22]. To address these challenges, the Pan-European Network for Care of Survivors after Childhood and Adolescent Cancer (PanCare) has developed the Survivorship Passport (SurPass), which includes a personalised treatment summary, survivorship care plan, and plain language information for CCSs and their relatives [14]. Several versions of SurPass have been developed and validated as part of previous EU projects [18, 23].^1^ The latest version, SurPass v2.0 (hereafter referred to as SurPass), is currently being optimised in the PanCareSurPass (PCSP) project (https://www.pancaresurpass.eu). This new, digital version allows for semi-automated data input and interoperability between different care providers, empowering CCSs to take the lead in their own LTFU care.

The present study builds on the PCSP pre-implementation study, which used a semi-structured online survey to identify barriers and facilitators [24, 25]. We conducted Open Space meetings to further explore and address these issues [26]. The primary objective of this study is to explore stakeholders’ perspectives on the implementation of SurPass in six LTFU centres across Europe, with a specific focus on the barriers and facilitators identified in the online survey. The overall aim of this study is to formulate practical recommendations to overcome significant barriers and capitalise on facilitators to support the successful implementation of SurPass. Based on our findings, we will develop general guidelines for the implementation of SurPass.

## Methods

### Participants

SurPass will be implemented and evaluated in six survivorship care centres in six countries, including Austria (Children’s Cancer Research Institute St. Anna Kinderkrebsforschung), Belgium (Katholieke Universiteit Leuven), Germany (Universität zu Lübeck), Italy (Istituto Giannina Gaslini), Lithuania (Viesoji Istaiga Vilniaus Universiteto Ligonine Santaros Klinikos), and Spain (Fundación para la Investigación del Hospital Universitario la Fe de la Comunidad Valenciana). For this study, the participating stakeholder groups associated with each of the participating centres included HCPs, CCSs, care managers, IT specialists, and others (e.g. cancer registry members, parent association representatives, and data managers). Each meeting was attended by stakeholders invited by the centre hosting the meeting. The aim was to assemble 20–75 stakeholders per centre.

### Study design: Open Space meetings

Open Space is a qualitative research method for organising and conducting productive and creative discussions in which participants are invited to self-organise, create, and manage the agenda [26]. Open Space meetings are not moderated. Open Space is based on four principles: (1) Whoever comes is the right person, (2) Whatever happens is the only thing that can happen, (3) Whenever it starts is the right time, and (4) When it is over, it is over. In addition, the Law of Two Feet encourages participants to move into discussions where they can contribute effectively [26]. Participants’ roles include ‘owners’ who initiate topics, ‘bumblebees’ who move between topics, and ‘butterflies’ who practice quiet reflection. Participants are free to change roles during the discussion. A total of six online Open Space meetings were held in the participating PCSP partner centres, with each meeting exclusively attended by participants from the respective institution.

### Procedure

The first author (IdB) received training in the Open Space method from the training centre ‘Het Eerste Huis’ (https://www.heteerstehuis.nl). As the Open Space meetings were conducted in the local languages, the author also trained local leaders from the PCSP centres who were responsible for facilitating the Open Space meetings and inviting participants by e-mail. Due to the aftermath of the COVID-19 pandemic, the Open Space meetings were held online via Zoom. The invitation included a description of the purpose of the Open Space meeting, the date and time, a Zoom user guide, and a list of potential discussion topics based on the barriers and facilitators identified in the online survey [24, 25]. All meetings started with an introduction to the SurPass and an explanation of the Open Space approach by IdB, followed by the agenda creation process. If a participant had an idea or a question they wanted to discuss, they posted a topic, along with their name, on a virtual bulletin board that included multiple time slots. Once all participants had a chance to originate a topic, they could choose which and how many discussions they wanted to take part in. The local leaders of the centres determined the number of discussion rounds. During each round, participants were free to move around between the virtual breakout rooms where the discussions took place simultaneously. After each round, there was a short break of 10–20 min. Tech hosts were available throughout the meeting to provide technical support as needed, although no significant problems arose. The owners of the discussion topics were responsible for recording the minutes in a digital discussion summary form, which included the participants and the main conclusions of the discussion. Personal data collected from participants was limited to what was relevant for data analysis, i.e. how many stakeholders from each stakeholder group were involved. No names, contact details, or other personal identifiers were collected before or during the Open Space meetings. In all six centres, ethical review boards were consulted about the need for their ethical approval. Formal ethical approval was only required and obtained in Spain. Participants from Spain signed an informed consent form before the Open Space meeting, which was then kept locally. After the Open Space meetings, the discussion summary forms were translated into English by the centres and sent to the Princess Máxima Center in the Netherlands for analysis.

### Data analysis

#### Barriers and facilitators

The study outcomes were the statements made during the group discussions, which were recorded on the digital discussion summary form. All statements from the summary forms were imported into Microsoft Excel. A three-level thematic analysis was then carried out based on a conceptual framework of relevant action fields. Thematic analysis is a method for identifying, analysing, and reporting on themes within a qualitative data set [27]. Our three-level thematic analysis involved level 1 barriers and facilitators; level 2 the action fields Care, ELSE (E-ethical, L-legal, S-social, E-economical), and Information & IT (as previously determined in the PCSP project agreement); and level 3 specific themes. Level 1 indicated whether a statement from the summary form represented a barrier or a facilitator. At the second level, barriers and facilitators were categorised within one of the action fields, reflecting the multiple levels of implementation that are considered relevant within the PCSP project (Care, ELSE, and Information & IT). In the latter, information refers to data and knowledge, while IT refers to the tools and systems used to collect, process, and manage this information. At the third level, barriers and facilitators were linked to matching sub-themes reflecting the content of the statements made by participants during the Open Space meetings (e.g. involved staff, data protection, or system integration). The majority of the sub-themes assigned in this study corresponded to the themes assigned to the barriers and facilitators identified in the online survey [24, 25], but where certain statements did not correspond to these pre-defined sub-themes, additional sub-themes were created based on the semantic content of the statements [27]. Overall, a combination of deductive and inductive coding was used, as we started deductively with the barrier/facilitator distinction and the five action areas, and then inductively created new sub-themes where necessary for the third level classification. To optimise interrater reliability, two authors (IdB and EH) independently coded the barriers and facilitators and reached consensus on the themes in cases of disagreement. A representative from each centre verified the final results. In order to provide a concise summary, we have included in the results (Table 2) only those barriers and facilitators that were identified in two or more centres. A complete list of all barriers and facilitators can be found in Supplementary Table 1.

#### Recommendations

The author group formulated general recommendations for the implementation of SurPass in the participating centres and for potential future centres interested in implementing SurPass. These recommendations were derived from a synthesis of the barriers and facilitators identified in the Open Space meetings and the previously described survey [24, 25] and the expertise of the author group. First, each specific barrier or facilitator identified in the Open Space meetings and the survey underwent a content evaluation to assess its general applicability to SurPass implementation in all centres. If a particular barrier or facilitator was deemed relevant, we ensured that it was appropriately rephrased and integrated into the final set of recommendations. Second, beyond the insights from the identified barriers and facilitators, the author group formulated further recommendations based on expert opinion. The recommendations were written in a generic way to ensure their applicability across all PCSP centres, allowing each centre to tailor the implementation of the recommendation to its specific context.

## Results

### Characteristics of participants and Open Space meetings (Table 1)

**Table 1:** Characteristics of participants and Open Space meetings

Country	CCSs	HCPs	Care managers	IT specialists	Others	Total participants	Total rounds	Time per round	Total topics
Austria	2	6	0	6	2^a^	16	2	30 min	3
Belgium	1	9	3	1	13^b^	27	2	25 min	7
Germany	8	12	0	1	15^c^	36	3	25 min	13
Italy	3	7	0	7	3^d^	20	1	40 min	3
Lithuania	5	12	1	6	1^e^	25	2	15 min	7
Spain	6	10	1	9	0	26	3	20 min	11
Total	25	56	5	30	34	150	13	275 min	44

^a^Austria: 1 CCSs advocate and 1 project manager;

^b^Belgium: 2 legal service workers, 2 cancer association representatives, 3 parent association representatives, and 6 parents of CCSs;

^c^Germany: 6 parents of CCSs, 3 foundations representatives, 3 data managers, 2 cancer association representatives, and 1 ethics expert;

^d^Italy: 1 biostatistician, 1 data manager, and 1 administrator;

^e^ Lithuania: 1 parent association representative.

*CCSs* childhood cancer survivors, *HCPs* healthcare providers, *IT* information and technology.

The final research population (*n* = 150) consisted of 25 CCSs, 56 HCPs, 5 care managers, 30 IT specialists, and 34 others. Across centres, there were 16 participants in Austria, 27 in Belgium, 36 in Germany, 22 in Italy, 25 in Lithuania, and 26 in Spain. All Open Space meetings took place in April 2022. The number of discussion rounds varied from one round (*n* = 1) to two (*n* = 3) or three (*n* = 2), with all rounds lasting between 15 and 40 min (mean = 26 ± 9). The total number of topics discussed in each round ranged from 3 to 13 (mean = 7 ± 4).

### Barriers to the implementation of SurPass (Table 2)

**Table 2:** Barriers and facilitators for the implementation of the SurPass v2.0: all centres combined

Action fields	Themes	Barriers	Facilitators
Care	Organisational context	• Lack of LTFU care and network cooperation^b,c,e,f^	• Standardised and centralised LTFU care^b,c,d,e,f^ • SurPass should be included in transition towards adult care^c,d^
	Knowledge (about care)	• Lack of knowledge of long-term effects among HCPs^c,f^ • Screening and LTFU care recommendations unknown^c,f^	
	SurPass content		• SurPass should include a psychological component^a,b,c,d^ • Involvement of a contact person for further questions regarding SurPass^b,c^ • Contact option for psychosocial care within SurPass would be helpful^b,c^ • Psychosocial components in SurPass should be optional to activate (not standard)^b,c^ • SurPass should be positively oriented^b,c^
Ethical	SurPass access	• Uncertainty about who should be able to access SurPass (e.g. parents, HCPs, and insurance companies)^a,b,c,f^ • Uncertainty about how impaired CCSs can access SurPass^b,c^	• SurPass should not be accessible for insurance providers^b,f^ • SurPass access rights must be granted by the patient^b,c,e^
	Data protection		SurPass should have an option for CCSs to view and conceal sensitive information^a,b,c,e^
	Anxiety in CCSs	• SurPass could cause stress/anxiety/fear^b,c^ • SurPass could lead to over-concern^b,c^ • SurPass could lead to ‘catastrophising’ (every minor thing could be seen as a serious illness)^b,c^	
Legal	Secondary use of data		• Importance of distinction between collecting data for treatment or research objectives^b,c,f^ • Synchronisation of care and research data^c,f^
Information & IT	SurPass usability	• Uncertainty about how to lower the threshold to use SurPass^b,c^ • Uncertainty about how SurPass data can be used in other countries^b,c,f^	• Usability of SurPass abroad^c,d^
	System integration	• Uncertainty about the integration of different data formats^c,f^ • Uncertainty about how the SurPass is updated^c,d,f^ • Uncertainty about system integration between national and European health systems^b,c,d,f^	• Automatic filing of SurPass from hospital information systems^a,b,c,d,e,f^ • Automatic transfer of data from SurPass to HIS^a,b,c,d,e,f^
	Knowledge about SurPass		• Promotion material should provide information about SurPass in simple language^b,c,d,f^
	SurPass format		• SurPass should include all common languages to choose from^c,d,f^

This table only includes barriers and facilitators identified by two or more centres. The complete list of all barriers and facilitators can be found in Supplementary Table 1.

^a^Austria (St. Anna Kinderkrebsforschung)

^b^Belgium (Katholieke Universiteit Leuven)

^c^Germany (UMC Mainz)

^d^Italy (Istituto Giannina Gaslini)

^e^Lithuania (Viesoji Istaiga Vilniaus Universiteto Ligonine Santaros Klinikos)

^f^Spain (Fundación para la Investigación del Hospital Universitario la Fe de la Comunidad Valenciana)

*CCSs* childhood cancer survivors, *HCPs* healthcare providers, *(HL7) FHIR* Fast Healthcare Interoperability Resources.

**Fig. 1:**
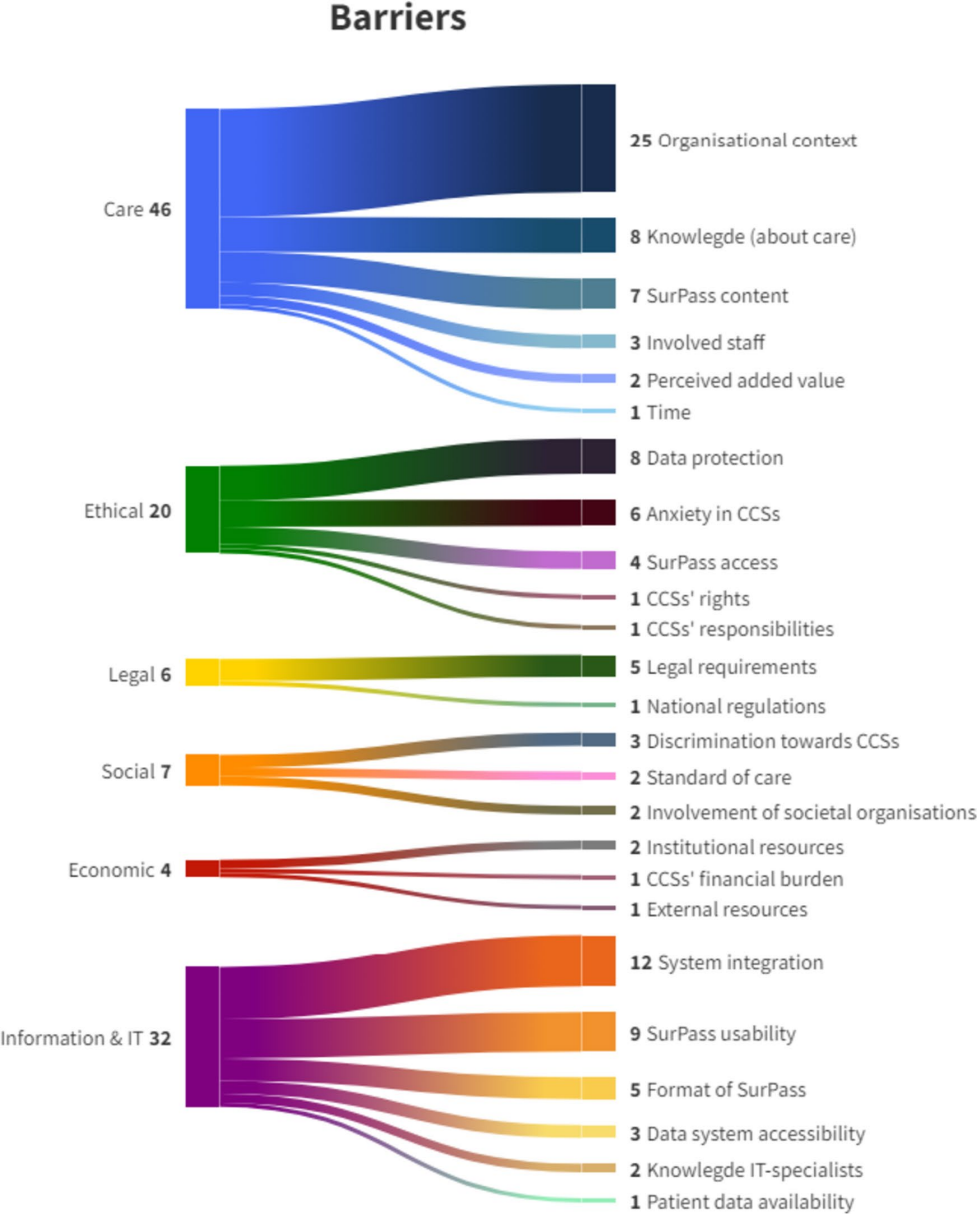
Schematic representation of identified barriers (*n* = 115) categorised into action fields (Care, Ethical, Legal, Social, Economic, Information & IT) and themes. *Note.* The numbers in this figure represent the frequency of occurrence of the action fields (left) and the sub-themes (right)

In total, 115 barriers were identified and subdivided into the action fields Care, ELSE, Information & IT, and related themes (Fig. 1). For the Care-related action field, the main barriers included the lack of LTFU care and the lack of cooperation/collaboration between LTFU centres and/or HCPs involved in LTFU care, which was mentioned in 4/6 countries. In addition, participants from Germany and Spain recognised the lack of knowledge among HCPs and CCSs about long-term effects as a barrier. For the ethical part, uncertainties about access to SurPass were expressed in 4/6 countries. For instance, participants were unsure whether insurance companies or parents/caregivers would ultimately have access to SurPass. Moreover, participants from Belgium and Germany raised questions about how CCSs with an impairment/disability would be able to access SurPass (e.g. in case of blindness or other sensory or cognitive problems). In both of these countries, another worry included potential stress, anxiety, or fear that SurPass could cause in CCSs because of the constant confrontation with their cancer history and its consequences. A prominent barrier in the Information & IT action field involved the uncertainty of 5/6 countries about how to integrate SurPass into their electronic health information systems (EHIS). Participants from Germany, Italy, and Spain also indicated being uncertain about how to update SurPass. Furthermore, Belgium, Germany, and Spain disputed how CCSs could use SurPass when they travel to other countries. Only participants from Spain identified evident legal and social issues. With regard to the legislation, the Spanish participants were uncertain about how to agree on the exchange of information in SurPass. Social barriers identified during the Open Space meeting in Spain highlighted the need for CCSs to have equity regarding insurance policies and the lack of involvement of societal organisations. Moreover, Austria and Spain both identified the lack of financial resources to be able to implement the SurPass as an economic barrier.

### Facilitators to the implementation of SurPass (Table 2)

**Fig. 2:**
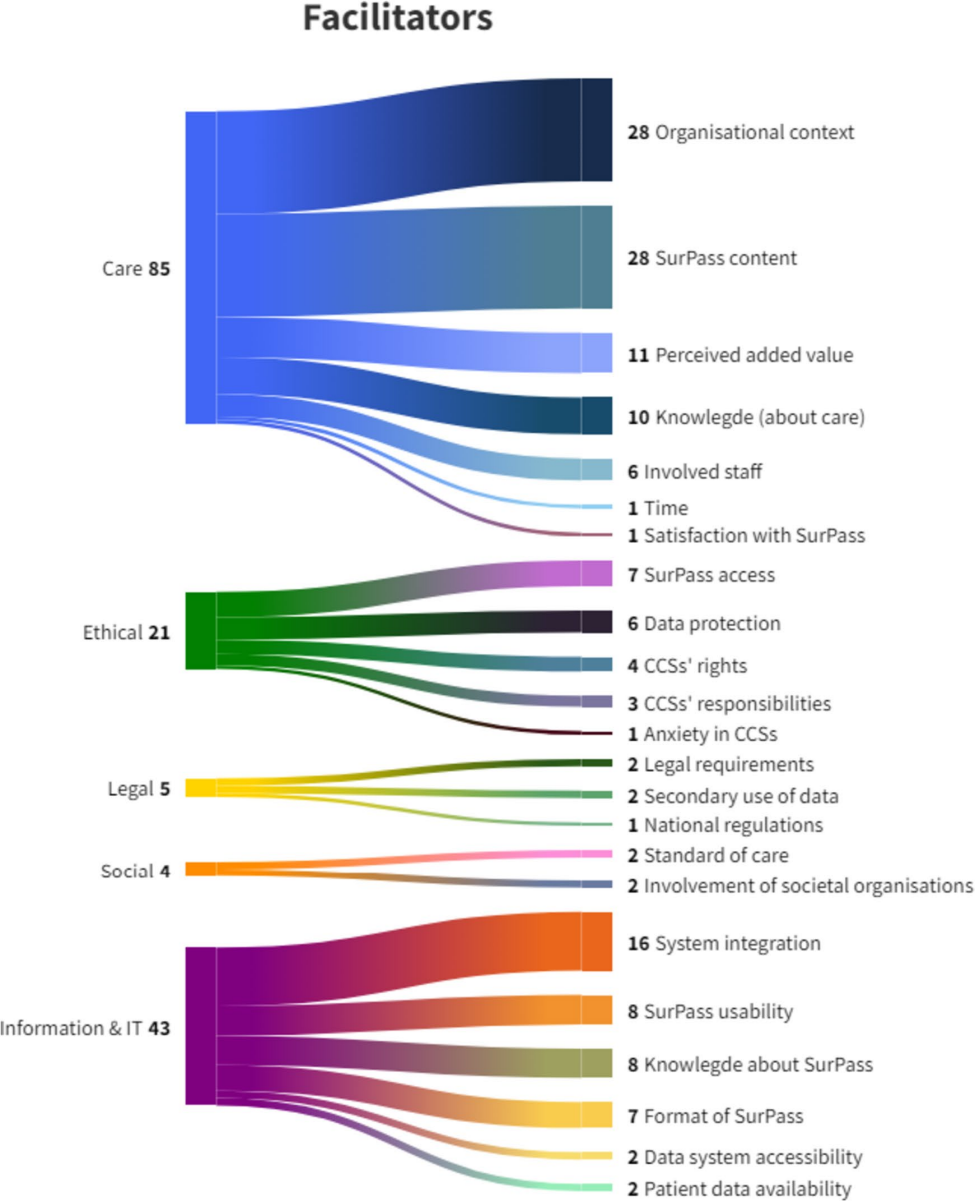
Schematic representation of identified facilitators (*n* = 159) categorised into action fields (Care, Ethical, Legal, Social, Information & IT) and themes. *Note.* The numbers in this figure represent the frequency of occurrence of the action fields (left) and the sub-themes (right)

A total of 159 facilitators have been identified (Fig. 2). With regard to the Care action field, 5/6 countries concluded that standardised and coordinated LTFU care is critically important. For instance, there should be systems and routines that ensure high-quality cooperation, communication, and shared responsibilities among HCPs. Participants from Belgium and Germany expressed their desire to have a contact point for questions or concerns concerning LTFU care. When looking at facilitators regarding the structure and layout of SurPass, 4/6 countries indicated that SurPass should include medical as well as psychological components. In addition, Belgium and Germany stated that SurPass should be positively oriented (e.g. by emphasising that regular physical activity can help maintain a healthy weight, strengthen muscles and bones, and improves overall physical and mental health) and should not limit its focus to health risks and disease only. Lastly, in 2/6 Open Space meetings, participants expressed that SurPass should be included in the transition from paediatric to adult care settings. Concerning the ethics-related action field, Belgium and Spain mentioned SurPass not being accessible for insurance companies as a facilitator. Belgian, German, and Lithuanian participants wanted CCSs to be granted SurPass access and alteration rights. These countries and Austria also opted to build a function in SurPass that allows to conceal sensitive information, such as psychological effects caused by earlier treatment. In addition, Belgian participants wanted to grant SurPass access to informal care providers or counsellors when necessary. When looking at the Legal action field, participants from 3/6 countries agreed that it is important to be transparent about collecting SurPass data for either treatment objectives and/or research objectives. Furthermore, Italian participants wanted SurPass to be a recognised as an official document at every national level. A facilitator within the Social action field concerned the availability of parent associations as an intermediary between CCSs and HCPs, coined by the Spanish participants. Finally, regarding the Information & IT-related action field, participants from each of the six countries emphasised the importance of semi-automatic transfer of electronic health records to SurPass. Relatedly, the automatic filing of the information in SurPass was considered an important facilitator mentioned in all countries. To aid the integration of data into different EHIS, German participants suggested the appointment of data managers. Moreover, participants from Germany, Italy, and Spain highlighted the importance of SurPass’ availability in all European languages. To educate CCSs about SurPass and how to use and interpret the information provided, participants from 4/6 countries suggested supplementing the SurPass with concise, simplified information, such as brochures and picture books. No facilitating factors were identified within the Economic action field.

### Recommendations for the implementation of SurPass (Table 3)

**Table 3:** Recommendations for the implementation of the SurPass v2.0

Action fields	Themes	Recommendations
Care	Organisational context	• Use the internationally developed protocols and guidelines to implement LTFU care (e.g. from the International Late Effects of Childhood Cancer Guideline Harmonization Group ^a^, PanCareSurFup, and PanCareFollowUp projects [13, 18, 20]) • Generate an interdisciplinary transition from paediatric to adult care • Provide multidisciplinary LTFU care including case managers and specialised HCPs • Join the PanCare network
	Knowledge (about care)	• Develop and disseminate knowledge regarding LTFU care (surveillance), e.g. by hosting information events (such as PanCare meetings) • Provide LTFU care training for CCSs
	SurPass content	• Include a contact option for LTFU care, including mental healthcare, within SurPass (e.g. telephone number, e-mail address, website for information) • Include mental healthcare information in SurPass
Ethical	Data protection	• Add an option in SurPass for CCSs to view and hide sensitive information
	SurPass access	• Make clear decisions about which parties (e.g. HCPs, insurance providers, family members) have access to SurPass data in accordance with CCSs • Decide together with impaired CCSs how best to access SurPass (e.g. together with a parent, guardian, or caregiver)
	CCSs’ rights	• Investigate the feasibility of allowing CCSs to decide what information is shared in their SurPass
	Anxiety in CCS	• Adequately explain to CCSs the potential disadvantages of receiving a SurPass before letting them decide whether to receive one • Adequately explain the SurPass during LTFU care consultation • Provide psychological support to CCSs to reduce stress, anxiety, and fear
Legal	Data storage	• Investigate how long SurPass data will be retained and communicate this to the CCSs
	Secondary use of data	• Clearly communicate to CCSs when SurPass data will be used for LTFU care and when it will be used for research purposes
Social	Involvement of societal organisations	• Stimulate survivors to join the European Network of Youth Cancer Survivors (EU-CAYAS-NET)^b^ • Involve regional and global organisations (e.g. Childhood Cancer International and PanCare) to maximise collaboration regarding SurPass implementation
Economic	Institutional resources	• Advocate for policies and funding (e.g. at (non)governmental organisations, and charities) to support SurPass implementation
	Cost-effectiveness	• Make use of the prediction model (which is currently under development) to assess the costs and benefits of SurPass
Information & IT	SurPass usability	• Develop a set of standardised guidelines outlining the steps and best practices for implementing SurPass in different healthcare settings • Ensure that SurPass has an intuitive and user-friendly interface • Conduct SurPass user testing and gather feedback from users to make necessary improvements
	System integration	• Use HL7 FHIR for (semi)automated data exchange and interoperability of health data systems
	Knowledge about SurPass	• Provide promotional material with information about SurPass in plain language
	SurPass format	• Ensure that SurPass is available in all European languages

^a^The International Late Effects of Childhood Cancer Guideline Harmonization Group (IGHG) represents a common vision and integrated strategy for the surveillance of chronic health problems and subsequent cancers in childhood, adolescent, and young adult cancer survivors. An overview of all IGHG publications and recommendations can be found on www.ighg.org.

^b^The mission of EU-CAYAS-NET is to establish a European network of young cancer survivors as well as an knowledge centre and interactive social networking platform that will enable cancer survivors to advocate for their needs and rights.

*CCSs* childhood cancer survivors, *HCPs* healthcare providers, *HL7 FHIR* Health Level 7 Fast Healthcare Interoperability Resources, *LTFU* long-term follow-up, *SurPass* Survivorship Passport.

We have formulated 27 recommendations that relate to the Care (*n* = 8), Ethical (*n* = 7), Legal (*n* = 2), Social (*n* = 2), Economic (*n* = 2), and Information & IT (*n* = 6) action fields. For example, we suggest using the internationally developed protocols and guidelines for implementing LTFU care to address the lack of LTFU care and network cooperation across centres. In addition, we recommend that everyone involved in survivorship care (e.g. HCPs, policy makers, survivors, and relatives) to join the PanCare network to work with the European community to increase awareness and research on childhood cancer survivors. An example recommendation from the Ethical action field is to add an option in SurPass for CCSs to view and hide sensitive information. For the Legal action field, we recommend clearly communicating to CCSs when SurPass data is used for LTFU care and when it is used for research purposes. Secondly, we recommend investigating how long SurPass data is retained and communicating this to the CCSs as well. For the Social action field, we recommend involving societal organisations such as the European Network of Youth Cancer Survivors (EU-CAYAS-NET), Childhood Cancer International Europe (CCI Europe), and PanCare to maximise collaboration. In terms of the Economic action field, we recommend advocating for policies and funding to increase institutional resources and using the prediction model (currently being developed in the PCSP project) to assess the costs and benefits of SurPass for each centre. Finally, an example of a recommendation from the Information & IT field is the use of HL7 Fast Healthcare Interoperability Resources (FHIR) for (semi)automated data exchange and interoperability of health data systems.

## Discussion

In this Open Space study, we identified 115 barriers and 159 facilitators for the implementation of SurPass v2.0. Based on these findings, we developed 27 general recommendations to support the implementation of SurPass in each of the participating PCSP centres.

The first main barrier was related to the Care action field and included the lack of LTFU care and network cooperation. This barrier can be overcome by capitalising on one of the key recommendations: providing standardised and coordinated LTFU care. Insights from the ongoing PanCareFollowUp project could be used to aid in the implementation of person-centred LTFU care in various healthcare centres across Europe [23]. The *PanCareFollowUp Care Intervention* includes crucial components such as person-centred care, increased awareness of late effects, shared decision-making, and empowering survivors to seek medical or psychosocial help if needed, support adapting a healthy lifestyle [18, 23]. SurPass can contribute to this process by providing a comprehensive summary of treatment history and personal recommendations for surveillance and prevention. This information can help healthcare providers tailor survivorship care to the individual needs of each survivor, which is a key component of person-centred care [16]. In the future, SurPass can ensure that survivors receive appropriate care and support by providing healthcare providers with a clear understanding of the survivor’s treatment history and potential risks of late effects, including evidence-based guidelines for follow-up care and plain language summaries of late effects.

The second main barrier related to the Ethical action field and included uncertainty regarding who could access SurPass data. To overcome this barrier, we recommend making clear decisions about which parties have access to SurPass data in accordance with CCSs. Relatedly, participants expressed a desire for CCSs to have control over their SurPass content, including determining what information is shared and with whom. To address this, we suggest allowing CCSs to conceal sensitive information, in accordance with the ‘right to be forgotten’ in Article 17 of the General Data Protection Regulation (GDPR) (gdpr.eu). This can be facilitated by implementing a feature that enables CCSs to manage their own data sharing preferences within SurPass. Allowing CCSs to control their SurPass content can help to increase their sense of ownership and involvement in their healthcare, which could in turn lead to better engagement and adherence to recommended surveillance and prevention measures.

The last main barrier to implementing SurPass was related to Information & IT and included uncertainty about how to integrate SurPass into national, regional, or local EHIS. Correspondingly, we recommend automatic transfer and filing of SurPass data. To facilitate semi-automatic data transfer between EHIS and SurPass, the internationally recognised HL7 FHIR standard for data access and exchange could be utilised [24]. HL7 FHIR provides a standardised interface for the exchange of electronic health information. Furthermore, to ensure efficient integration, clear agreements on data retention and coding systems are recommended. This would allow for consistent and standardised use of SurPass data across different healthcare settings. By agreeing on a standardised coding system, healthcare providers can easily share information and ensure that SurPass data is accurately recorded and efficiently used. Moreover, establishing clear retention policies can help ensure that SurPass data is stored securely and is accessible when needed, while avoiding unnecessary data retention.

Furthermore, providing concise and simplified information in the form of brochures and picture books, as well as including both medical and psychological components in SurPass, was identified as important facilitators. Specifically, the participants highlighted the importance of providing CCSs and their parents/guardians with additional resources that supplement the textual information provided in SurPass. These resources should be easily understandable and visually appealing, such as brochures and picture books, which can aid in the comprehension of complex information about cancer treatment and late effects. Additionally, the participants emphasised the importance of incorporating both medical and psychological components in SurPass to ensure a holistic approach to LTFU care. SurPass can support this by providing recommendations for both medical and psychological support. Overall, these facilitators would contribute to a more user-friendly and accessible SurPass while improving the care and well-being of CCSs.

Because barriers and facilitators from similar studies may also be applicable to SurPass, we compared our findings with a systematic literature analysis on barriers and facilitators related to the implementation of eHealth tools similar to SurPass such as survivorship care plan applications [28]. Similar barriers include limited knowledge of the tool, lack of information on tool accessibility, fear of extra workload for HCPs, and uncertainty about data security. Correspondingly, important facilitators include stakeholder involvement in tool development, user-friendliness, improved communication between patients and HCPs, and integration into clinical routine care. Another recent study identified data security, accessibility, and the lack of monitoring and management coordination as significant barriers to eHealth tool implementation [29]. Positive attitudes towards eHealth tools and health data exchange between primary and secondary health services were important facilitators. The findings of the current study underscore and add to the currently available literature.

The strength of our study was the use of the innovative Open Space research methodology, which allowed participants to identify and discuss relevant topics related to SurPass implementation in depth, despite the short time frame. Moreover, this methodology facilitated interaction between stakeholder groups that would not typically collaborate, which resulted in a wide range of discussion topics and diverse perspectives. Additionally, the involvement of participants from six different European countries further contributed to a broad and comprehensive understanding of the barriers and facilitators to the implementation of SurPass. Yet, some limitations of the current study must be acknowledged. First, only five care managers participated in the Open Space meetings, possibly leading to an underrepresentation of their experiences and views on the implementation of SurPass. The lack of discussion topics regarding the Economic action field could be explained by the scarcity of managers as well. Second, although online Open Space meetings enabled participants to tune in more easily than face-to-face meetings, the online Zoom environment may have caused fewer interactions between participants during the discussions.

In conclusion, this study identified three key barriers in the areas of Care, Ethical considerations, and Information & IT. The primary barriers identified were the lack of LTFU care programmes and network cooperation, uncertainty regarding SurPass accessibility, and challenges with respect to the integration of SurPass into EHIS. To address these barriers, important recommendations include using the internationally developed protocols and guidelines to implement LTFU care, making clear decisions about which parties have access to SurPass data in accordance with CCSs, and facilitating (semi)automated data transfer and filing using HL7 FHIR. These recommendations can provide valuable guidance to PCSP centres and other countries considering the adoption of SurPass v2.0. Ultimately, the implementation of SurPass v2.0 is expected to enhance LTFU care and improve the quality of life for CCSs globally.

## Supplementary Information

Below is the link to the electronic supplementary material.

Supplementary file1 (PDF 303 KB)

## Data Availability

Study participants did not consent to data sharing outside the PanCareSurPass project. Data access is therefore limited to national and international supervisory authorities. Upon request, the study protocol can be made available (please send an e-mail to i.a.e.debeijer-3@prinsesmaximacentrum.nl).

## Abbreviations

CCSs: Childhood and adolescent cancer survivors
EHIS: Electronic health information systems
ELSE: Ethical, legal, social, and economic
FHIR: Fast Healthcare Interoperability Resources
HCPs: Healthcare providers
LTFU: Long-term follow-up
PCSP: PanCareSurPass
SurPass: Survivorship Passport

## Footnotes

1 The European Network for Cancer Research in Children and Adolescents (ENCCA; SurPass v1.0), the European Expert Paediatric Oncology Reference Network for Diagnostics and Treatment (ExPO-r-Net; SurPass v1.0.1; https://www.expornet.eu/), PanCareSurFup (SurPass v1.1; https://www.pancaresurfup.eu/), and PanCareFollowUp (SurPass v1.2; www.pancarefollowup.eu).
