# Correction: Barriers and facilitators to the implementation of a new European eHealth solution (SurPass v2.0): the PanCareSurPass Open Space study

**DOI:** 10.1007/s11764-024-01547-w

**Published:** 2024-03-11

**Authors:** Ismay A. E. de Beijer, Emma C. Hardijzer, Riccardo Haupt, Desiree Grabow, Julia Balaguer, Edit Bardi, Adela Cañete Nieto, Audronė Ciesiūniene, Vanessa Düster, Anna-Liesa Filbert, Hannah Gsell, Monika Kapitančukė, Ruth Ladenstein, Thorsten Langer, Monica Muraca, Selina R. van den Oever, Sofie Prikken, Jelena Rascon, Maria Teresa Tormo, Anne Uyttebroeck, Gertrui Vercruysse, Helena J. H. van der Pal, Leontien C. M. Kremer, Saskia M. F. Pluijm, the PanCareSurPass consortium

**Affiliations:** 1 Princess Máxima Center for Pediatric Oncology, Heidelberglaan 25, 3584 CS Utrecht, The Netherlands; 2 IRCCS Istituto Giannina Gaslini, Genoa, Italy; 3 Division of Childhood Cancer Epidemiology, German Childhood Cancer Registry, Institute of Medical Biostatistics, Epidemiology and Informatics (IMBEI), University Medical Center, of the Johannes Gutenberg University Mainz, Mainz, Germany; 4 Hospital Universitario y Politécnico La Fe, Valencia, Spain; 5 St. Anna Children’s Hospital, Vienna, Austria; 6 Department of Paediatrics and Adolescent Medicine, Johannes Kepler University Linz, Kepler University Hospital, Linz, Austria; 7 Vilnius University Hospital Santaros Klinikos, Vilnius, Lithuania; 8 St. Anna Children’s Hospital and Children’s Cancer Research Institute, Department of Studies and Statistics for Integrated Research and Projects, Department of Paediatrics, Medical University of Vienna, Vienna, Austria; 9 CCI Europe, Vienna, Austria; 10 Universitatsklinikum Schleswig-Holstein, Campus Lubeck, Lübeck, Germany; 11 University Hospitals Leuven, KU Leuven, Louvain, Belgium; 12 Instituto de Investigación Sanitaria La Fe, Valencia, Spain; 13 University Medical Center Utrecht, Wilhelmina Children’s Hospital, Utrecht, the Netherlands; 14 Department of Pediatrics, Emma Children’s Hospital, Amsterdam UMC, University of Amsterdam, Amsterdam, The Netherlands

**Correction: Journal of Cancer Survivorship**

10.1007/s11764-023-01498-8

In the original publication of the article, on page 2, something has gone wrong with the layout, i.e., the text is unreadable.

The authors Gertrui Vercruysse and Anne Uyttebroeck should have affiliation 10 (KU Leuven) instead of 11 (Lubeck Germany).

The above correction has been applied in this paper.

