# Supplementary material for: Barriers and facilitators to the implementation of a new European eHealth solution (SurPass v2.0): the PanCareSurPass Open Space study
Source: J Cancer Surviv. 2023 Nov 28;19(2):659–71. doi: 10.1007/s11764-023-01498-8 (PMC11926050; doi:10.1007/s11764-023-01498-8)
Supplement: Supplementary file 1 — Supplementary file1 (PDF 303 KB) [file 11764_2023_1498_MOESM1_ESM.pdf]

Supplementary Table 1. All barriers and facilitators for the implementation of SurPass.

Action Fields	Themes	Barriers	Facilitators
**Care**	Organisational context	• There are too many guidelines for LTFU care for CCSs [a]<br>• Lack of good guidance on child-adult care transition [b]<br>• Lack of monitoring LTFU attendance [b]<br>• Uncertainty about how CCSs will be contacted to introduce SurPass [b]<br>• Management of diseases takes much time and resources for CCSs and their relatives [c]<br>• Lack of LTFU care and network cooperation [b,c,e,f]<br>• GP should be linked to LTFU care HCPs [c]<br>• Lack of interdisciplinary contact points for CCSs for psychological late effects [c]<br>• Lack of LTFU care after 5 years post-treatment in Germany [c]<br>• Lack of a standard way of how paediatric oncologists work [c]<br>• Uncertainty if a single centre for LTFU care is feasible [c]<br>• Lack of checklist/guideline on how to best communicate late effects [c]<br>• Requirement of a referral for a medical specialist is needed [e]<br>• Referrals should be clearer [e]<br>• Lack of monitoring of health conditions [e]<br>• Lack of single functioning centre for LTFU care [e]<br>• Lack of shared care institutions [e]<br>• Lack of follow-up visits for late effect screening only [f]<br>• Too little CCSs for each quota of primary care doctors [f]<br>• LTFU care included in research instead of health system [f]	• Importance of psychosocial aspects to be discussed with the psychologist/social worker [a]<br>• CCSs should be introduced to SurPass at the end of treatment [b]<br>• Standardised and centralised LTFU care [b,c,d,e,f]<br>• CCSs trying to manage the LTFU care (e.g. medication and consults) themselves experience difficulty regarding documentation [c]<br>• Less complicated requirements to obtain LTFU care documents [c]<br>• SurPass should be included in transition towards adult care [c,d]<br>• Delivery of SurPass through LTFU clinic [c]<br>• Clinic needs to support informing the CCSs or relatives and hand over a SurPass [c]<br>• Provide care structures [c]<br>• Health insurance companies should be involved [c]<br>• Psychological late effects should be standardised [c]<br>• Transition from paediatric to adult care should be interdisciplinary [c]<br>• HCPs should prepare SurPass [c]<br>• Contact person for late effects should be included [c]<br>• Communication with GPs should be improved [c]<br>• There should be ownership in the management of information and LTFU care [c]<br>• Involvement of case managers in LTFU care [c]<br>• Implementation of adult LTFU care centres [c]

	• Lack of coordinators between HCPs [f]<br>• Lack of recommendations/LTFU care for patients with chronic diseases [f]<br>• Lack of psychological follow-up of CCSs [f]	• Use of video consultations in LTFU care [c]<br>• Coordination within in LTFU care [c]<br>• Uncertainty about what should be done to improve LTFU care until SurPass is implemented [c]<br>• Creation of a data base of HCPs in LTFU care [c]<br>• Building strong local teams with HCPs who feel locally connected and are in close exchange with the LTFU outpatient clinic [c]<br>• Importance of the transition of paediatric care to adult care [d]<br>• LTFU should be included nationally and in the cancer plan of each community [f]<br>• Knowledge sharing within primary healthcare [f]<br>• LTFU consultation in each community in the portfolio of care services [f]<br>• Creation of protocols by the regional ministries to increase resources and obligate LTFU care [f]<br>• Notifications within digital health systems [f]
Time	• Workload too high when the HCPs has to validate the correctness of data sent in FHIR format to CINECA [d]	• Clear timeline of when to include which information in the SurPass [c]
Involved staff	• Lack of a contact point for young adult CCSs [c]<br>• Doctors for adult CCSs need to be involved in monitoring [e]<br>• Concerns about increase of workload of HCPs [f]	• Late effects specialists should take the lead in LTFU care [b]<br>• Involvement of a contact person for further questions regarding SurPass [b,c]<br>• Nationwide list of HCPs for LTFU care should be developed [c]<br>• Private practice HCPs should be included in LTFU care [c]<br>• Involvement of the family physician is of great importance [f]<br>• Hiring data managers for cases prior to EHR [f]

	Knowledge (about care)	• Lack of knowledge of long-term effects among HCPs [c, f]<br>• Screening- and LTFU care recommendations unknown [c, f]<br>• Uncertainty about what the psychological late effects are [c]<br>• Uncertainty about what tools exist for LTFU care [c]<br>• Uncertainty about when to inform CCSs about late effects [c]<br>• Lack of health education for families [f]<br>• Lack of training for different HCPs [f]<br>• Lack of knowledge in insurance companies regarding late effects [f]	• CCSs should be updated about relevant novel research findings [b]<br>• Education about LTFU care for CCSs [c]<br>• Sharing information about late effects as soon as possible [c]<br>• Oncology education for medical student [c]<br>• LTFU care education for oncological paediatricians [c]<br>• Information events about LTFU care for GPs [c]<br>• Providing informative material/educational options for CCSs about late effects [c]<br>• Awareness about LTFU care should be raised by the GPs [d]<br>• Semi-annual debriefing meetings between specialists to share information [e]<br>• Importance of disease knowledge in children < 16 years [f]
	Experience		• SurPass is seen as a tool to be aware of wellness and check-ups for CCSs [d]
	Perceived added value	• Uncertainty among centres outside St. Anna if SurPass will add any extra value to LTFU care [a]<br>• Uncertainty which stakeholders will benefit from SurPass [a]	• University Hospital Graz would like to have SurPass implemented [a]<br>• Relief of future possible illnesses – SurPass helps to remember following consultations etc. [b]<br>• Digital SurPass could be useful when centralised with no additional administrative burden [b]<br>• Digital SurPass useful for young CCSs because they will not be dependent on parents [b]<br>• SurPass can inform CCSs about the risks, long-term effects and screenings [b]<br>• SurPass is expected to be a helpful tool in the transition towards adult care [c]

		• SurPass is perceived to be helpful to reduce the need to explain late effects [d]<br>• Surpass could act as a referral to specialist [e]<br>• Convenient for doctors to see aggregated information in SurPass for further treatment decisions [e]<br>• Useful to have information concentrated in SurPass [e]<br>• SurPass considered useful for research to improve treatment [e]
Satisfaction with Surpass		• SurPass implementation would benefit from a sound evaluation[a]
SurPass content	• Uncertainty on what psychosocial information is needed or wanted in SurPass [a]<br>• SurPass is only focused on health risks instead of prevention [b]<br>• Lack of information about minor ailments in SurPass [b]<br>• Lack of information about how CCSs can improve their lives themselves (e.g. exercise or lifestyle) [b]<br>• Uncertainty about when information is entered into SurPass [b]<br>• Uncertainty about how to determine which information should be included in SurPass [c]<br>• Uncertainty about the need for oncological and other medical information [f]	• Simple but detailed info in SurPass can stop the "catastrophising" [b]<br>• Information in SurPass is formulated differently for CCSs and HCPs [b]<br>• Preventive screening for possible serious illnesses: automatically planned & arranged by SurPass [b]<br>• HCPs easily accessible for CCSs via single point of contact in SurPass [b]<br>• SurPass should include positive messages/experiences from more experienced CCSs [b]<br>• SurPass should include a mentorship option for CCSs [b]<br>• LTFU care should include medical as well as psychological components [b]<br>• SurPass could involve a prevention component [b]<br>• Important to include psychosocial care aspects in LTFU care [b]<br>• SurPass could include guidelines for psychosocial topics [b]

	• Psychosocial components in SurPass should be optional to activate (not standard) [b, c]<br>• Nuance is important in SurPass [b]<br>• Contact option for psychosocial care within SurPass would be helpful [b,c]<br>• Standardised medication plan in the SurPass is desirable [c]<br>• SurPass should be a dynamic document linked (and updated) to the development of the child [c]<br>• New findings must also be recorded in the SurPass after 30 years [c]<br>• SurPass should include a psychological component [a,b,c,d]<br>• SurPass should include plain language information for young/cognitive impaired CCSs [c]<br>• SurPass should be positively oriented [b,c]<br>• A structure in which all information on the course of the disease and LTFU care is brought together and can be found again without difficulty is seen as desirable [c]<br>• Importance to distinguish what kind of data and relation to the cancer phase should be entered in SurPass [c]<br>• SurPass should include the invisible disabilities and overall welfare of CCSs [d]<br>• Updates of recommendations based on individual information is needed [e]<br>• Special function within SurPass for continuously monitoring health complications [e]<br>• Complete treatment summery converted directly into SurPass would be useful [e]

			• Notifications for reaching patient milestones or appointments [f]<br>• Notifications notifying hospitals to call the CCSS for appointments [f]
**Ethical**	SurPass access	• Uncertainty about who should be able to access SurPass (e.g. parents, HCPs and insurance companies) [a,b,c,f]<br>• Uncertainty about who receives SurPass [b]<br>• Uncertainty about how impaired CCSs can access SurPass [b,c]<br>• Uncertainty about who has access to add data into SurPass [f]	• SurPass should not be accessible for insurance providers [b, f]<br>• Informal care should have access to SurPass [b]<br>• Counsellors for CCSs with limited autonomy should have access to SurPass [b]<br>• SurPass access rights must be granted by the patient [b,c,e]<br>• SurPass is issued at the "5-year follow-up" and then handed over to CCSs or relatives [c]<br>• Accessibility of SurPass should be well prepared and implemented [c]<br>• Visibility of Surpass for all doctors is permitted by CCSs [e]
	Data protection	• Uncertainty what will happen to the data in SurPass [a]<br>• Information can only be registered via the GDA (health authority) - only the GDA is allowed to register in ELGA [a]<br>• Uncertainty if CCSs allow SurPass data that benefits others to be shared [a]<br>• Data protection of European citizens differs outside the European Union [b]<br>• Uncertainty about data retention [b]<br>• Validation of data cannot be done automatically [d]<br>• Importance of data security and data protection [f]<br>• Importance of the geographical location of the data and related security measures [f]	• Informed consent by CCSs is needed to add sensitive information in SurPass [b]<br>• SurPass should have an option for CCSs to view and conceal sensitive information [a,b,c,e]<br>• Need of a control authority to ensure the patient has been informed and that the SurPass has been handed over [c]<br>• The patients' consent form could include an option to agree with transferring Surpass to ESPBI [e]<br>• Data should be codified [f]

		• Using patient identifiers for information use outside SurPass (e.g. SIP and DNI) [f]	
	CCSs' rights	• Uncertainty about what happens when parents do not inform their child about SurPass [c]	• Co-design/co-creation involving CCSs is necessary [a]<br>• CCSs' requests should be included into SurPass [a]<br>• Autonomy of younger CCSs should be considered [b]<br>• CCSs would like to see what ESPBI documents look like [e]
	Anxiety in CCSs	• SurPass could cause stress/anxiety/fear [b,c]<br>• SurPass could lead to over-concern [b,c]<br>• SurPass could lead to "catastrophising" (every minor thing could be seen as a serious illness) [b,c]<br>• Anxiety can cause people not to use SurPass [b]<br>• Uncertainty about when it is the right time to mention late effects [c]<br>• Anxiety caused by the lack of appropriate LTFU care [f]	• Dose and repeat the information about late effects to prevent the feeling of being overwhelmed [c]
	CCSs' responsibilities	• The need for CCSs to know they have to take responsibility for SurPass [f]	• Care program should be positively oriented and controllable by CCSs themselves [b]<br>• SurPass will bring empowerment to CCSs because of their own coordination of care [c]<br>• Advice for CCSs on taking responsibility [c]
**Legal**	Legal requirements	• Importance of site hosting server regarding legal issues [b]<br>• Communication tailored to CCSs instead of parents when legal age is reached [b]<br>• Lack of obligation to inform CCSs about late effects [c]<br>• Uncertainty about how to agree on the exchange of information [f]	• Informed consent is included into ELGA and should be in SurPass [a]<br>• SurPass should become accessible when CCSs transfer to adult care [c]

		• CCSs should have reached the legal age or age 16 to receive SurPass [f]	
	Secondary use of data		• Importance of distinction between collecting data for treatment or research objectives [b,c,f]<br>• Synchronisation of care and research data [c, f]
	National regulations	• Strict law regulation- tracking only up to 18 years [e]	• SurPass should be a recognised document at the national level [d]
**Social**	Standard of care	• Need for a clearly defined psychosocial list developed by CCSs at European level [a]<br>• Uncertainty about how to involve young adults with cancer [c]	• CCSs would like to receive e-mails regarding LTFU care from the clinic [c]<br>• Creation of LTFU care units with minimum standards [f]
	Involvement of societal organisations	• Lack of involvement of political institutions [f]	• Importance of the Parents' Federation to create pressure on LTFU care [f]<br>• Availability of parents' association as an intermediary between CCSs and HCPs [f]<br>• Involve global/European organisations to defend insurance equity of CCSs [f]
	Discrimination towards CCSs	• The name "SurPass" could lead to stigmatisation [b]<br>• Difficulties and differences in insurance for CCSs [f]<br>• The need for CCSs to be able to opt for the same insurance policies as other citizens [f]	
**Economic**	Institutional resources	• Further financial support is needed for implementation of SurPass in Austria [a]<br>• Concerns about the budget to be able to finalise SurPass project [f]	
	External resources	• Health insurance funds must finance SurPass implementation (Innsbruck) [a]	

	CCSs' financial burden	• CCSs' fear that insurers' knowledge of their situation will lead to a higher financial burden [f]	
**Information & IT**	Knowledge IT specialists	• Uncertainty on how the FSE can be kept updated [d]<br>• Uncertainty about how to possibly revise the electronic medical record structure [d]	
	Data system accessibility	• Uncertainty about how it is ensured that different file formats stay readable and accessible [c]<br>• No restriction of visibility of some or part of medical documents in ESPBI system [e]	• Data access from GP/paediatrician needed [c]<br>• Possibility to make the patient information in VULSK ELI system "sensitive" [e]
	Patient data availability	• Uncertainty about when data will be put in SurPass [c]<br>• Incompleteness of data due to missing key attributes [d]	• Including medication in SurPass is perceived to have no technical difficulties [a]<br>• Fixed date for creation and delivery is needed [c]
	SurPass usability	• Uncertainty if CCSs who have been opted out of ELGA also receive SurPass [a]<br>• Uncertainty if CCSs can add data into SurPass themselves [a]<br>• Uncertainty about how to lower the threshold to use SurPass [b,c]<br>• Administrative tasks for CCSs could lead to not using SurPass [b]<br>• Uncertainty about what should be done if CCSs refuse the SurPass [c]<br>• Uncertainty on how to update SurPass regarding psychological late effects [c]<br>• Uncertainty about how SurPass data can be used in other countries [b,c,f]<br>• Uncertainty if SurPass applies to young adult CCSs [c]<br>• Doubts regarding the need for internet to access SurPass [f]	• Cooperation of software producers, technical hospital team and developers is necessary [a]<br>• Pilot implementation of SurPass is planned for St. Anna [a]<br>• Implement a SurPass pilot in every PCSP centre [a]<br>• SurPass should address young CCSs and parents together [b]<br>• Usability of SurPass abroad [c,d]<br>• Joint meetings between IT and HCPs in order to test the coding of the live data [d]<br>• After discharge CCSs should still have access to SurPass [f]<br>• Traceability of what is written in SurPass and by whom [f]

System integration	• Different HIS systems in Austria - adaptations necessary for each centre [a]<br>• COZO, KUL differs from EPD [b]<br>• Uncertainty about the integration of different data formats [c, f]<br>• Uncertainty about how, when and by whom data is entered in SurPass [c]<br>• Data format not standardised across HIS [c]<br>• Uncertainty on how to integrate the SurPass with the FSE (which is a document of the PNNR) [d]<br>• Documents from the hospital can only be sent to the ESF by PDF format [d]<br>• Current development of the ESF in the PNRR which currently provides for the CDA2 format, while the project provides for FHIR [d]<br>• Uncertainty about how the SurPass is updated [c,d,f]<br>• Uncertainty about system integration between national and European health systems [b,c,d,f]	• Pilot for linking all HIS in Austria [a]<br>• Every hospital needs to set up a CDA to have common basis for linking all HIS [a]<br>• Austria is working on implementation of a common CDA to finish within 1-2 years [a]<br>• Linking SurPass and Cancer Registries could avoid duplication [b]<br>• The possibility of combining SurPass with the Health Card [c]<br>• Automatic filing of SurPass from HIS [a,b,c,d,e,f]<br>• Automatic transfer of data from SurPass to HIS [a,b,c,d,e,f]<br>• FHIR for data exchange and networking of clinics has to be used [c]<br>• HL7 FHIR offers possibility to generate data to GP [c]<br>• In the future documents of the citizen will be visible to the health professionals in Liguria (EHR) [d]<br>• The application formats the standardised documents in CDA2 [d]<br>• Development of software to make PDF files available to the ESF [d]<br>• Integration of multiple platforms to use SurPass [f]<br>• Integration with EHR [f]<br>• Standardisation of data [f]<br>• Facilitating the software and data collection correctly [f]
Knowledge about SurPass	• Uncertainty about who should be contacted about SurPass use (CCSs/parents?) **[b]**<br>• Uncertainty on how SurPass will be understandable for young children [c]	• Importance of disclosure regarding SurPass information to various HCPs [b]<br>• Consultation about SurPass should be provided [b]<br>• Send information about and together with SurPass [c]

		• Promotion material should provide information about SurPass in simple language [b,c,d,f]<br>• Information brochures to explain SurPass for older CCSs [c]<br>• Pixie-books for younger CCSs to explain SurPass [c]<br>• Education for HCPs about SurPass and its importance to CCSs [e]<br>• Learning how to use SurPass for HCPs and CCSs [f]
SurPass format	• Uncertainty about how blind CCSs can access the data [c]<br>• Uncertainty about what language should be used in SurPass for migrated CCSs [c]<br>• Uncertainty about a suitable target medium (folder, stick, PDF) [c]<br>• Uncertainty about the (time) structure of information which enters SurPass [c]<br>• Uncertainty about who will make SurPass digital [d]	• The name SurPass could be changed to "care program" [b]<br>• Data should be available as a PDF document rather than in paper [c]<br>• Storing SurPass electronically as a PDF document is seen as positive [c]<br>• SurPass should include all common languages to choose from [c, d, f]<br>• SurPass could use a multilingual approach (national language vs English) [c]<br>• SurPass should also still be available on paper [c]<br>• Bilingual double SurPass [c]<br>• Surpass should be an official document [d]<br>• SurPass should include the possibility to use the QR code/ link directly with a PDF that CCSs can open to consult the legislation [d]

[a] Austria (St. Anna Kinderkrebsforschung), [b] Belgium (Katholieke Universiteit Leuven), [c] Germany (UMC Mainz), [d] Italy (Istituto Giannina Gaslini), [e] Lithuania (Viesoji Istaiga Vilniaus Universiteto Ligonine Santaros Klinikos) and [f] Spain (Fundación para la Investigación del Hospital Universitario la Fe de la Comunidad Valenciana). Abbreviations: CCSs; childhood and adolescent cancer survivors, CINECA; supercomputing centre for scientific research in Italy, COZO; Collaboratief Zorgplatform (Collaborative Care Platform), DNI; Documento Nacional de Identidad (National Identity Document), EHR; electronic health records, ELGA; electronic health system in Austria, EPD; elektronisch patiëntendossier (electronic patient file), ESF; Italian Healthcare System, ESPBI; Electronic Health Services, EU; Europe, FHIR; Fast Healthcare Interoperability Resources, FSE; Fascicolo Sanitario Elettronico (Electronic Health Record), GDA; health

authority, GPs; general practitioners, HCPs; healthcare professional, HIS; Hospital Information Systems, KUL; Katholieke Universiteit België, LTFU; long-term follow-up, PNNR; Piano Nazionale di Ripresa e Resilienza (National Recovery and Resilience Plan), SIP; Session Initiation Protocol, St. Anna; St. Anna Kinderkrebsforschung, SurPass; Survivorship Passport.
